# Low-Affinity NMDA Receptor Antagonist Hemantane in a Topical Formulation Attenuates Arthritis Induced by Freund’s Complete Adjuvant in Rats

**DOI:** 10.34172/apb.2024.002

**Published:** 2023-07-19

**Authors:** Elena Ivanova, Alexander Matyushkin, Alexandra Sorokina, Svetlana Alexeeva, Irina Miroshkina, Kirill Kachalov, Tatyana Voronina, Andrey Durnev

**Affiliations:** ^1^Laboratory of Psychopharmacology, FSBI Zakusov Institute of Pharmacology, Moscow, Russia.; ^2^Laboratory of Drug toxicology, FSBI Zakusov Institute of Pharmacology, Moscow, Russia.

**Keywords:** Freund’s Adjuvant, Inflammation, NMDA receptor, Rats, Topical formulation

## Abstract

**Purpose:**

N-methyl-D-aspartate (NMDA) receptors that are expressed by T-cells modulate T-cell proliferation, cytotoxicity and cell migration toward chemokines. Several studies have shown an anti-inflammatory effect of NMDA receptor antagonists. This study compares the effect of the noncompetitive low-affinity NMDA receptor antagonist N-(2-adamantyl)-hexamethyleneimine hydrochloride (hemantane) in a topical formulation (gel) with the cyclooxygenase (COX) inhibitor diclofenac in a topical formulation (gel) in rats with arthritis induced by Freund’s Complete Adjuvant (FCA).

**Methods:**

On day 14 after an FCA injection into the left hind paw, rats with contralateral hind paw edema were selected for further investigation (29/65). They were treated with 5% hemantane gel or 1% diclofenac gel applied locally to hind paws daily for 2 weeks starting 14 days after the FCA injection. Rats with arthritis were examined hind paw edema, hyperalgesia, and motor deficits; their body weight and hematological parameters were recorded. The rats were euthanized on day 28, followed by histological examination of the ankle joint (HE stain).

**Results:**

Rats with arthritis exhibited hind paw inflammation and hyperalgesia, motor deficits, changes of hematological parameters, reduced weight gain and spleen hypertrophy. Histological examination of the ankle joint revealed degenerative-dystrophic lesions of the cartilaginous tissue, proliferative inflammation of the synovium, edema and lymphocytic/macrophage infiltration of periarticular tissues. Hemantane gel reduced hind paw edema, pain, motor deficits and histological signs of inflammation; its effect was comparable to diclofenac gel.

**Conclusion:**

Hemantane gel alleviates FCA-induced arthritis in rats, and its effect is comparable to diclofenac gel.

## Introduction

 The prevalence of musculoskeletal conditions increased by 63% in 1990-2019, and approximately 1.71 billion people globally suffer from musculoskeletal disorders. Of these, arthropathy exhibited the most dramatic growth. Between 1990 and 2019, the prevalence of osteoarthritis and rheumatoid arthritis (RA) increased by 114% and 106% respectively.^1^ Due to population growth and aging, the number of people with musculoskeletal conditions and related disabilities is rapidly increasing. Long-term therapy of such conditions must be safe in addition to being effective, ensuring that side effects of therapy do not impact the quality of life. For example, an updated algorithm of the European Society for Clinical and Economic Aspects of Osteoporosis, Osteoarthritis and Musculoskeletal Diseases (ESCEO) for knee osteoarthritis management strongly recommends topical non-steroidal anti-inflammatory drugs (NSAIDs) as cyclic add-on analgesia in Step 1 for patients with knee osteoarthritis who are still symptomatic after the use of Step 1 background therapy (chronic symptomatic slow-acting drugs), and prior to the use of oral NSAIDs. Delaying the use of oral NSAIDs is particularly important in patients with osteoarthritis and gastrointestinal, cardiovascular or renal comorbidities.^2^

 Topical NSAIDs were developed to improve the safety and tolerability of oral NSAIDs by decreasing systemic exposure and targeting affected tissues more directly.^3^ The anti-inflammatory and analgesic effects of NSAIDs are primarily driven by the inhibition of cyclooxygenase (COX) and subsequent prevention of prostaglandin (PG) biosynthesis from arachidonic acid.^3-5^ It was shown that levels of PGE2 and leukotriene (LT) LTB4, arachidonic acid derivatives, are higher in the joints of patients with osteoarthritis than in healthy individuals.^6,7^ Similarly to PG, LTB4 and cysteinyl LTs are potent mediators of inflammation, causing increased activation, recruitment, migration and adhesion of immune cells. Inhibition of COX by NSAIDs can cause alternative processing of arachidonic acid via the 5-lipoxygenase pathway, resulting in increased production of proinflammatory LTs.^8^

 Ionotropic glutamate receptors (iGluRs), in particular N-methyl-D-aspartate (NMDA) receptors, could be a potential target for topical anti-inflammatory and analgesic drugs for musculoskeletal conditions. IGluRs are localized on unmyelinated and myelinated axons in the periphery^9,10^ and the number of sensory axons containing iGluRs increases during inflammation.^11^ IGluRs are expressed by various types of immune cells, primarily T-cells, and produce immune effects upon direct binding of glutamate or its agonists/antagonists.^12^ NMDA receptor antagonists inhibit antigen-specific T-cell proliferation and cytotoxicity, as well as cell migration toward chemokines.^13^ A course of intraperitoneal memantine, an NMDA receptor antagonist, but not metabotropic glutamate receptor antagonists (R,S)-1-aminoindan-1,5-dicarboxylic acid or kynurenic acid, reduces synovitis and the frequency of erosions in mice with collagen-induced arthritis.^14^

 In the present study, we evaluated the effect of the noncompetitive low-affinity NMDA receptor antagonist N-(2-adamantyl)-hexamethyleneimine hydrochloride (hemantane)^15,16^ in a topical formulation compared to the COX inhibitor diclofenac in a topical formulation in rats with T cell-dependent arthritis induced by Freund’s Complete Adjuvant (FCA).

## Material and Methods

###  Animals

 The study was performed on 260-300 g mature white outbred male rats, which were obtained from the Stolbovaya animal breeding facility (Federal Medical-Biological Agency, Russia). The rats were housed under controlled environmental conditions (20-24 °C, 45-65% RH) with unrestricted access to food and water using a complete diet of extruded pellets, with a 12-hour light/12-hour dark cycle. The work was organized and conducted in compliance with GOST (Russian National Standard) 33216-2014, “Guidelines for accommodation and care of animals. Species-specific provisions for laboratory rodents and rabbits”; GOST (Russian National Standard) 33215-2014, “Guidelines for accommodation and care of animals. Environment, housing and management”; and Directive 2010/63/EU of the European Parliament and of the Council of 22 September 2010 on the protection of animals used for scientific purposes. The experimental protocol was approved by the Biomedical Ethics Committee, V.V. Zakusov Institute of Pharmacology (Protocol No. 01 of Jan. 31, 2020).

###  Reagents

 Hemantane gel 5% was developed and supplied by the Laboratory of Finished Dosage Forms, V.V. Zakusov State Institute of Pharmacology. Diclofenac gel 1% (Hemofarm, Serbia) was used as a comparator drug. FCA was obtained from Sigma-Aldrich Co. (St Louis, MO, USA).

###  Experimental protocol

 Adjuvant arthritis was induced by the administration of 0.1 mL of FCA into the plantar surface of the left hind paw^17^ of outbred rats. On day 14 after the injection of FCA, contralateral hind paw edema was measured as an increase of the metatarsus and the ankle joint diameter vs. baseline (day 0). The contralateral hind paw edema developed in only 29 out of 65 animals. These animals were chosen for further investigation and were divided into 3 groups: rats with adjuvant arthritis (AA group); rats with AA treated with 5% hemantane gel applied locally to hind paws (Hemantane gel group); and rats with AA treated with 1% diclofenac gel applied locally to hind paws (Diclofenac gel group).

**Figure 1:**
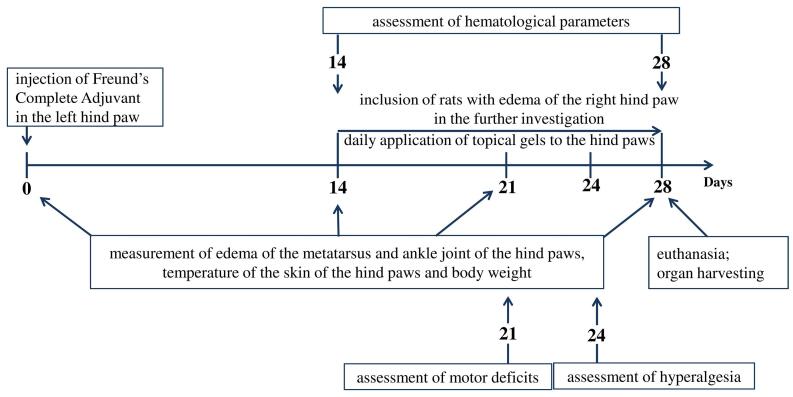
Experimental protocol

 Edema of the metatarsus and the ankle joint of the hind paws, body weight and skin temperature of the paws were recorded on days 0, 14, 21 and 28 after the FCA injection. Pain threshold was measured using the plantar test on day 24. Coordination of animals was measured in the rotarod test on day 21 after the FCA injection. Hematological parameters were assessed on day 14 and 28 (Figure 1).

###  Measurement of edema of the metatarsus and ankle joint

 The diameter of the left and right metatarsi and ankle joints was measured with a caliper before (day 0) and on days 14, 21, and 28 after the FCA injection. Hind paw edema was recorded as an increase in the diameter of the left and right metatarsi and ankle joints on day 14, 21 and 28 after the FCA injection relative to day 0 (before the FCA injection).

###  Measurement of hind paw skin temperature

 Hind paw skin temperature was measured with an infrared electronic thermometer (model DT-633, A&D medical, Japan) before (day 0) and on days 14, 21, and 28 after the FCA injection.

###  The rotarod test 

 The rotarod test (Ugo Basile for rats, Italy) was used for assessing motor deficits on day 21 after the FCA injection. A rotarod apparatus is a drum 6 cm in diameter separated into 4 equal portions by 5 discs 49 cm in diameter. The animals were pretrained by carrying out three trial runs at 5 rpm on the day of testing. Motor deficits were assessed with the drum accelerating from 10 to 30 rpm (at an acceleration of 1 rpm per 10 seconds). The time spent by the rats on the rotating rod was recorded (up to a maximum of 180 seconds).

###  The plantar test

 The plantar test (Hargreave’s Method, Ugo Basile, Italy) was used for assessing thermal hyperalgesia in rats on day 24 after the FCA injection. The rats were individually placed inside a transparent plastic chamber (20 × 10 × 14 cm) on the glass platform of the apparatus to acclimate for 15 minutes. An infrared heat source was focused on the middle of the plantar surface of the hind paws of the animals. The target temperature started at 40°С and increased slowly at a rate of 1 °C/second. The maximum exposure time was 20 seconds. Hind paw withdrawal latency was recorded.

###  Assessment of hematological parameters

 Hematological parameters were assessed on day 14 after the FCA injection (before treatment) and on day 28 (after 2 weeks of treatment). The rats were bled from the tail vein. An Abacus Junior VET hematology analyzer (Diatron, Austria) was used to record hematological parameters, namely, red blood cells, hemoglobin, hematocrit, platelets, plateletcrit, white blood cells (WBCs) and granulocytes. The WBC differential was determined using a computerized microscopy system (MECOS, Russia).

###  Spleen weight ratio assessment

 Spleen weight ratio was calculated using the following formula: weight ratio = organ weight (mg) / animal body weight (g).

###  Histological evaluation

 Joint tissue specimens were harvested from the right hind paws of each rat, fixed in 10% (V/V) neutral buffered formalin and decalcified. After that, samples were embedded in paraffin and cut into 5 μm sections. Hematoxylin and eosin staining was then performed using standard methods (40x/100x images).

###  Statistical analysis

 Statistical analysis of experimental results was performed using R v. 4.0.4. Normality was tested using the Shapiro–Wilk test followed by an evaluation of between-group equality of variances using Bartlett’s test. The Mann–Whitney U test was used for further statistical analysis because the distribution of data within the groups was non-normal and/or because of between-group differences of variances. Results were given as the median (1st and 3rd quartile). Between-group differences were considered statistically significant at *P* < 0.05.

## Results

###  Effect on hind paw edema

**Figure 2:**
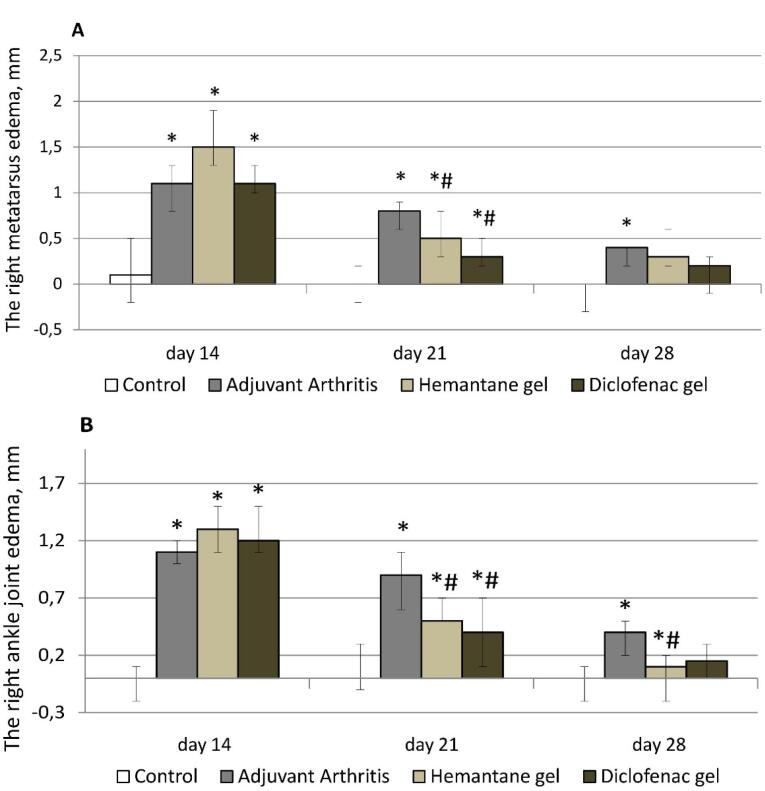
Effect of hemantane gel and diclofenac gel on right hind paw edema evoked by the FCA injection in rats indicated by an increase in the diameter of the right metatarsus (A) and ankle joint (B) compared with the values on day 0 (before the FCA injection). Data are expressed as median, Q1; Q3; * *P* < 0.05 vs. “Control group”; # *P* < 0.05 vs. “Adjuvant Arthritis group”; Mann–Whitney U test. Each group consists of 6-15 animals

 On day 14 after the injection of FCA (before external treatment), right hind paw edema was not significantly different across the groups with AA. Metatarsus diameter increased by 1.0-1.4 mm and ankle joint diameter increased by 1.1-1.4 mm compared with the control group (rats without AA, Figure 2).

 On day 21 after FCA injection, right hind paw edema was less pronounced in all groups with AA compared to day 14. In the groups treated with topical drugs, the reduction of edema was the strongest. After one week of therapy, the edema of the metatarsus and the ankle joint in the hemantane group decreased 3 and 2.6 times respectively compared to day 14, before therapy. In the hemantane group, edema of the right metatarsus improved by 37.5%, and edema of the right ankle joint improved by 44.4% compared to the AA group. The effect of 5% hemantane gel on right hind paw edema was not significantly different from the effect of 1% diclofenac gel. In the diclofenac group, edema of the metatarsus and the ankle joint decreased 3.7 and 3 times respectively compared to day 14. In the diclofenac group, edema of the right metatarsus was reduced by 62.5%, and of the right ankle joint, by 55.6% compared to the AA group. In the AA group, right hind paw edema decreased less than in both treatment groups: edema of the right metatarsus decreased by 27.3%, and of the right ankle joint, by 18.2% compared to day 14 (Figure 2).

 On day 28 after the FCA injection, right hind paw edema in all groups of rats with AA continued to improve. In the AA group, only the ankle joint remained significantly enlarged by 0.4 mm compared to the control group. Among the treatment groups, only 5% hemantane gel statistically significantly reduced ankle joint diameter by 0.3 mm compared to the AA group. There were no significant differences for right metatarsus or ankle joint diameters in the treatment groups relative to the control group (Figure 2).

**Figure 3:**
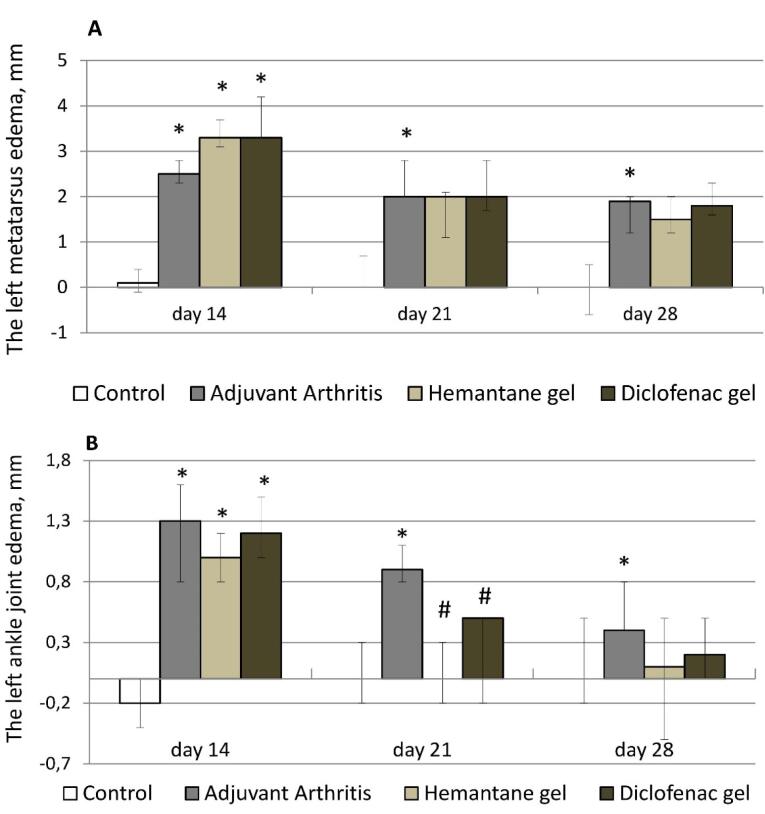
Effect of hemantane gel and diclofenac gel on left hind paw edema evoked by the FCA injection in rats indicated by an increase in the diameter of the left metatarsus (A) and ankle joint (B) compared with the values on day 0 (before the FCA injection). Data are expressed as median, Q1; Q3; * - *P* < 0.05 vs. “Control group”; #- *P* < 0.05 vs. “Adjuvant Arthritis group”; Mann–Whitney U test. Each group consists of 6-15 animals

 After the FCA injection, rats with right hind paw edema also developed left hind paw edema. Left metatarsus diameter increased by 2.4-3.3 mm, and ankle joint diameter increased by 1.2-2.0 mm compared to the control group (rats without AA). Left hind paw edema gradually decreased in all groups of rats with AA from day 14 to day 28 after the FCA injection. On day 21, metatarsal edema in the AA group decreased by 20% compared to day 14, and ankle joint edema, by 30.8%. On day 21, edema reduction was more pronounced in the hemantane and diclofenac groups. Edema of the left metatarsus decreased by 39.4% compared to day 14 in both groups. On day 21, edema of the left ankle completely resolved in the hemantane group and improved by 58.3% in the diclofenac group compared to day 14. On day 21, statistical differences between the AA group and treatment groups remained in ankle joint diameter only. On day 28, there was no measurable reduction in left metatarsal edema compared to day 21 either in the AA group or in the diclofenac group. On day 28, edema of the left metatarsus in the hemantane group improved by 25% compared to day 21, but the improvement was not statistically different from the AA group. On day 28, left ankle joint edema in the AA group was insignificant at 0.4 mm, a reduction of 55.6% compared to day 21. It almost completely resolved in the diclofenac group and was absent in the hemantane group (Figure 3).

 Additionally, hind paw skin temperature was recorded on days 14, 21 and 28 after the FCA injection. No differences between any of the study groups (the control group, the AA group, the hemantane group and the diclofenac group) were observed throughout the experiment at 31.1-32.0 °C on day 14, 29.0-30.4 °C on day 21 and 28.7-30.4 °C on day 28 after the FCA injection. A lack of difference in skin temperature between the control group and the other groups indicates the absence of acute inflammation in all groups.

###  Effect on motor deficits

**Figure 4:**
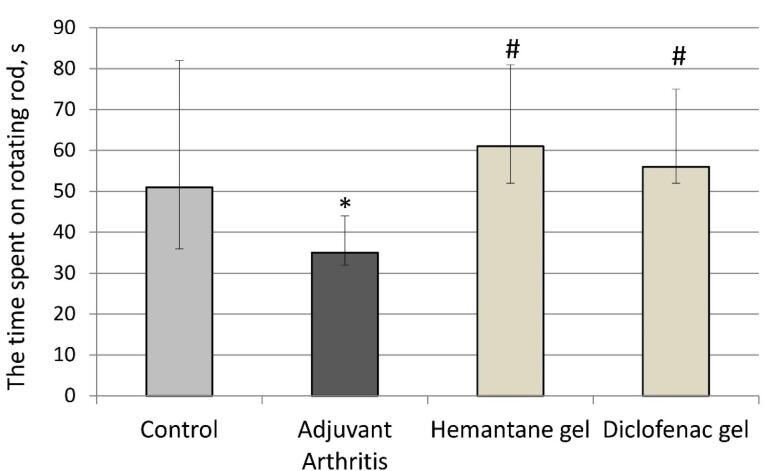
Effect of hemantane gel and diclofenac gel on motor deficits evoked by the FCA injection in rats (Median, Q1; Q3). * *P* < 0.05 vs. “Control group”; ^#^*P* < 0.05 vs. “Adjuvant Arthritis group”; Mann–Whitney U test. Each group consists of 6-15 animals

 Motor deficits were assessed using the rotarod test (Ugo Basile, Italy) on day 21 after the FCA injection. Rats with AA that did not receive any drugs exhibited motor deficits: the time spent on the rotating rod by the AA group decreased by 31.4% compared to the control group (p < 0.05). The gels under investigation reduced the severity of FCA-induced motor dysfunction. Hemantane gel and diclofenac gel increased the time spent on the rotating rod compared to the AA group by 74.3% and 60% respectively (Figure 4).

###  Effect on hyperalgesia

**Figure 5:**
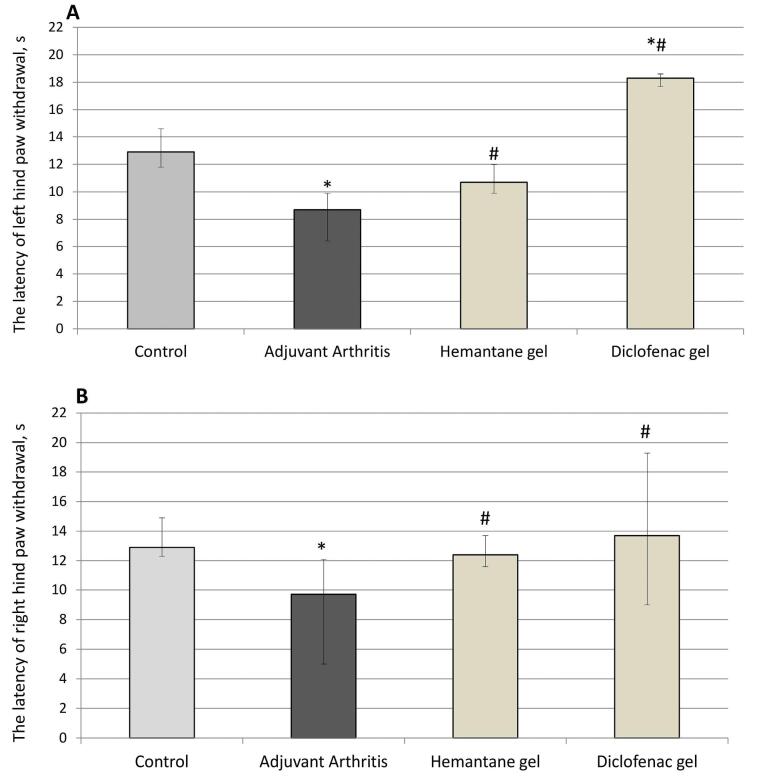
Effect of hemantane gel and diclofenac gel on hyperalgesia evoked by the FCA injection in rats indicated by the latency of left (4A) and right (4B) hind paw withdrawal in the plantar test (Median, Q1; Q3). *P < 0.05 vs. “Control group”; ^#^*P* < 0.05 vs. “Adjuvant Arthritis group”; Mann–Whitney U test. Each group consists of 6-15 animals

 Hyperalgesia was assessed in the plantar test (Ugo Basile, Italy) on day 24 after the FCA injection. The AA group exhibited hyperalgesia: paw withdrawal latency after the application of a painful stimulus to their plantar surface by radiant heat decreased by 32.6% for the left hind paw and by 24.8% for the right hind paw compared to the control group (*P* < 0.05). Hemantane gel and diclofenac gel reduced hyperalgesia in rats with AA. Hemantane increased the latency by 23.0% for the left hind paw and 34.0% for the right hind paw compared to the AA group (*P* < 0.05). Diclofenac gel was superior in this respect, increasing the latency by 106.7% in the left hind paw and 41.2% in the right hind paw compared to the AA group (*P* < 0.05) (Figure 5).

###  Effect on weight gain and mortality

 Rats with AA exhibited reduced weight gain compared to controls throughout the experiment (Table 1). On day 14 after the FCA injection, rats with AA gained 28.3-37.3% less weight than AA-free rats. On days 21 and 28, weight gain in the AA group was 32.4% and 31.0% less than in the control group. Hemantane gel and diclofenac gel had no significant effect on weight gain in rats with AA.

**Table 1:** Weight gain in rats with arthritis induced by Freund’s Complete Adjuvant

**Groups, number of animals in group on day 14**	**Weight gain relative to day 0**		
	**day 14, before treatment**	**day 21**	**day 28**
Control, n = 15	53 (41; 61)	71 (60; 86)	87 (74; 103)
Adjuvant Arthritis, n = 11	32 (26; 39)*	48 (45; 55)*	60 (53; 67)*
Hemantane gel, n = 8	37 (25; 43)*	39 (19; 54)*	67 (58; 80)*
Diclofenac gel, n = 10	33 (26; 52)	47 (39; 57)*	69 (37; 86)

* *P* < 0.05 vs adjuvant arthritis group, Mann–Whitney U test; data are expressed as median (Q1; Q3).

 Hemantane gel exhibited no toxic effects; in contrast, the toxicity of diclofenac gel was made evident by the death of 4 rats out of 10 in the relevant group while no animals died in the control or AA group.

###  Effect on hematological parameters

 On day 14 after the FCA injection (before treatment), a number of hematological parameters in rats with AA were altered compared to the control group (Table 2). In rats with AA, platelet count was elevated by 12.4%-28.2%, and plateletcrit, by 9.1%-31.8%. Granulocyte count was significantly increased in the AA group (by 57.8%) and in the hemantane group (135.6%). WBC count in the AA group was also increased (by 58.4%, *P* < 0.05). WBC differential in the treatment groups on day 14 showed a significant increase in segmented neutrophil percentage to 32.0-44.0% (i.e. by 33.3%-83.3%) compared to the control group (24.0%). In the AA group, the increase in segmented neutrophil percentage was not so pronounced at 18.8% vs. the Control group (*P* > 0.05). A compensatory decrease in lymphocyte percentage was observed in all groups with AA compared to the Control group: the reduction was 29.9%, 12.5% and 9.0% in the hemantane, diclofenac and AA groups respectively (Table 2).

**Table 2:** Hematological parameters of rats with arthritis induced by Freund’s Complete Adjuvant (FCA) on day 14 after the injection of FCA (before treatment)

**Parameters**	**Groups, number of animals in group on day 14**			
	**Control, n=15**	**Adjuvant Arthritis, n=11**	**Hemantane gel, n=8**	**Diclofenac gel, n=10**
Red blood cells, 10^12^/L	7.95 (7.66; 8.66)	8.67 (8.25; 9.10)	8.27 (8.04; 8.65)	8.17 (7.99; 8.72)
Hemoglobin, g/L	134.0 (123.0; 140.1)	137.5 (134.8; 139.8)	138.0 (137.0; 146.50)	141.0 (130.0; 146.0)
Hematocrit, %	43.04 (41.70; 47.09)	44.31 (43.88; 46.63)	43.69 (42.85; 46.05)	43.24 (41.99; 46.42)
Platelets, 10^9^/L	428.00 (389.50; 464.00)	481.00 (457.80; 552.80)*	548.50 (511.50; 587.00)*	511.00 (440.80; 525.20)*
Plateletcrit, %	0.22 (0.20; 0.25)	0.24 (0.24; 0.28)*	0.29 (0.26; 0.30)*	0.26 (0.23; 0.29)#
White blood cells, 10^9^/L	12.32 (0.65; 16.41)	19.52 (15.87; 21.37) *	17.32 (14.62; 19.46)	15.43 (14.45; 16.42)
Granulocytes, 10^9^/L	3.65 (3.22; 4.56)	5.76 (5.06; 6.57)*	8.60 (5.57; 9.93)*	5.70 (4.73; 6.17)
Stab neutrophils (%)	1.0 (0.5; 2.0)	1.0 (0.0; 1.0)	1.0 (1.0; 1.8)	1.0 (0.5; 1.0)
Segmented neutrophils (%)	24.0 (23.0; 29.0)	28.5 (23.5; 32.8)	44.0 (36.0; 49.0)*	34.0 (30.0; 40.5)*
Eosinophils (%)	1.0 (0.0; 1.0)	0.5 (0.0; 2.0)	0.0 (0.0; 1.5)	1.0 (0.0; 1.5)
Basophils (%)	0.0 (0.0; 0.0)	0.0 (0.0; 0.0)	0.0 (0.0; 0.0)	0.0 (0.0; 0.0)
Monocytes (%)	2.0 (2.0; 4.0)	4.0 (3.0; 4.0)	3.0 (3.0; 3.8)	3.0 (2.5; 3.0)
Lymphocytes (%)	72.0 (67.0; 72.5)	65.5 (63.0; 72.5)	50.5 (46.0; 58.0)*	63.0 (56.5; 64.0)*

* *P* < 0.05 vs. the control group, Mann–Whitney U test; data are expressed as median (Q1; Q3).

 On day 28 after the FCA injection, segmented neutrophil percentage in the AA group decreased by 28.1%, and lymphocyte percentage increased by 12.2% compared to day 14. Hemantane gel was associated with a significant reduction in segmented neutrophil percentage by 43.2% and a compensatory increase in lymphocyte percentage by 40.6% compared to day 14. Diclofenac gel did not significantly alter segmented neutrophil percentage compared to day 14 (before treatment). However, in the diclofenac gel group, hematocrit decreased by 7.6% compared to the AA group (Table 3).

**Table 3:** Hematological parameters of rats with arthritis induced by Freund’s Complete Adjuvant (FCA) on day 28 after the injection of FCA (after two weeks of treatment)

**Parameters**	**Groups, number of animals in group on day 28**			
	**Control, n=15**	**Adjuvant Arthritis, n=11**	**Hemantane gel, n=8**	**Diclofenac gel, n=10**
Red blood cells, 10^12^/L	9.09 (8.23; 9.35)	9.44 (8.77; 9.98)	8.94 (8.20; 9.39)	8.70 (8.60; 8.80)
Hemoglobin, g/L	137.00 (133.00; 141.50)	138.00 (134.00; 143.50)	136.50 (126.80; 141.80)	133.00 (127.50; 138.50)
Hematocrit, %	45.53 (40.89; 47.45)	47.38 (46.29; 49.63)	45.17 (40.30; 47.40)	43.78 (43.05; 44.62)#
Platelets, 10^9^/L	434.00 (399.00; 451.00)	412.00 (387.50; 441.20)	438.50 (382.50; 474.20)	495.00 (440.20; 548.80)
Plateletcrit, %	0.23 (0.20; 0.24)	0.21 (0.20; 0.22)	0.21 (0.19; 0.24)	0.26 (0.23; 0.28)
White blood cells, 10^9^/L	16.91 (14.28; 18.96)	17.27 (16.47; 20.29)	13.95 (12.99; 15.9)	16.75 (15.73; 18.67)
Granulocytes, 10^9^/L	4.34 (3.85; 5.20)	4.14 (3.71; 5.55)	4.76 (3.80; 5.37)	4.53 (3.96; 6.93)
Stab neutrophils (%)	1.0 (0.0; 1.0)	0.0 (0.0; 1.0)	0.5 (0.0; 1.8)	0.5 (0.0; 1.3)
Segmented neutrophils (%)	19.0 (16.5; 26.0)@	20.5 (17.3; 23.3)@	25.0 (21.3; 27.3)#*@	26.5 (19.8; 36.0)#*
Eosinophils (%)	0.0 (0.0; 1.0)	0.5 (0.0; 1.5)	0.5 (0.0; 1.0)	0.0 (0.0; 0.5)
Basophils (%)	0.0 (0.0; 0.0)	0.0 (0.0; 0.0)	0.0 (0.0; 0.0)	0.0 (0.0; 0.0)
Monocytes (%)	4.0 (3.0; 4.5)	4.0 (2.8; 6.0)	2.0 (2.0; 3.5)	4.0 (3.8; 5.0)
Lymphocytes (%)	74.0 (71.0; 77.5)	73.5 (69.8; 78.0)@	71.0 (69.5; 74.8)@	69.5 (58.0; 76.3)

* *P* < 0.05 vs. control group, Mann–Whitney U test; # *P* < 0.05 vs. adjuvant arthritis group, Mann–Whitney U test; @ *P* < 0.05 vs. day 14 after the injection of FCA, Mann–Whitney U test; data are expressed as median (Q1; Q3).

###  Effect on spleen weight ratio assessment

 The effect of 2 weeks of therapy on spleen weight and weight ratio (organ weight in mg/rat body weight in g) was assessed. Compared to the control group, spleen weight in the AA group increased by 19.6%, and spleen weight ratio, by 15.6%. Neither diclofenac gel nor hemantane gel affected spleen weight or weight ratio (Table 4).

**Table 4:** Spleen weight and weight ratio (organ weight in mg/rat body weight in g) in rats with arthritis induced by Freund’s Complete Adjuvant

**Groups**	**Spleen weight, mg**	**Spleen weight ratio**
Control	1453.0 (1287.5; 1638.0)	4.5 (4.2; 4.9)
Adjuvant Arthritis	1740.0 (1636.0; 2247.0)*	5.2 (4.6; 7.0) *
Hemantane gel	1351.5 (1228.5; 1610.0)	4.8 (4.1; 5.2)
Diclofenac gel	1521.0 (1246.0; 1769.0)	4.7 (4.2; 5.9)

* *P* < 0.05 vs. control group, Mann–Whitney U test.

###  Histological evaluation of the hind paw

**Figure 6:**
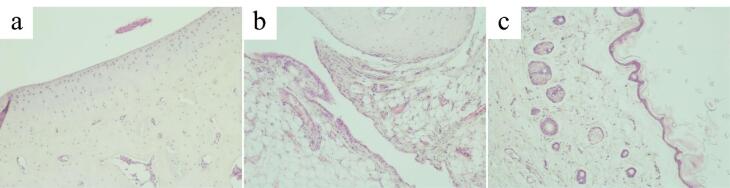
Articular cartilage, synovial membrane and periarticular tissues of the control group. (a). Articular cartilage of the ankle joint of the control group (original magnification х100). (b) Synovial membrane of the ankle joint of the control group (original magnification х100). (c) Periarticular tissue of the ankle joint of the control group (original magnification х100)

 Microscopic examination of the ankle joint of the control group revealed that tissues of the joint, the synovial membrane and periarticular tissues exhibited no signs of inflammation or destruction. Articular cartilage was divided into the superficial zone, the transitional, the deep zone and the calcified zone. Small chondrocytes in the superficial zone lay in the intercellular substance at a distance from each other and mainly had a flat shape, with hyperchromatic nuclei. The transitional zone was composed of randomly organized obliquely oriented fibrils. In the deep zone, chondrocytes with basophilic cytoplasm were arranged in columns perpendicular to the articular surface. The hyaline cartilage contained no nerves or blood vessels. The calcified zone, penetrated by capillaries, consisted of calcified cartilage with a more intensely colored intercellular substance and chaotically scattered groups of small hyperchromic chondrocytes (Figure 6a). From the inside, the articular surface was lined with an areolar type synovial membrane penetrated by blood and lymphatic vessels and nerve fibers. The synovial folds protruded into the joint cavity. Synovial cells were densely arranged, sometimes in several layers (Figure 6b). The periarticular tissue of rats in the control group exhibited no signs of inflammation (Figure 6c).

**Figure 7:**
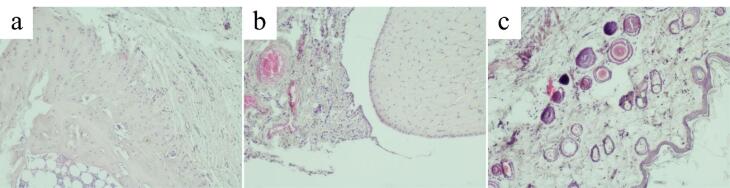
Articular cartilage, synovial membrane and periarticular tissues of rats with adjuvant arthritis (AA) induced by an injection of Freund’s Complete Adjuvant. (a) Articular cartilage of the ankle joint of the AA group (original magnification х100). (b) Synovial membrane of the ankle joint of the AA group (original magnification х100). (c) Periarticular tissue of the ankle joint of the AA group (original magnification х100). AA, adjuvant arthritis

 Joint tissues, synovial membrane and periarticular tissues of rats in the AA group exhibited morphological signs of chronic productive inflammation. Articular cartilage also exhibited signs of dystrophy and destruction. The zoning of articular cartilage was not clearly defined (Figure 7a).

 The articular surface had varying thickness. Boundaries of the articular surface were indistinct and often had an uneven, pitted surface. Small hyperchromic chondrocytes of the hyaline cartilage of the superficial zone were randomly dispersed in the intercellular substance. Chondrocytes of the deep zone mostly did not form columns. The calcified zone contained isolated sparsely scattered chondrocytes.

 Injury of the articular cartilage by FCA induced synovial hyperplasia. Synoviocytes were densely grouped. Lymphocytes and macrophages with an admixture of isolated polymorphonuclear leukocytes were found among the collagen fibers. The synovium was penetrated by many newly formed blood vessels and capillaries filled with blood cells (Figure 7b). In the periarticular tissue, signs of lymphomacrophage infiltration with an admixture of single polymorphonuclear leukocytes were observed (Figure 7c).

**Figure 8:**
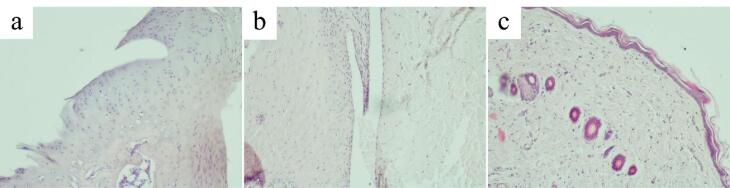
Articular cartilage, synovial membrane and periarticular tissues of rats in the hemantane group. (a) Articular cartilage of the ankle joint in the hemantane group (original magnification х100). (b) Synovial membrane of the ankle joint in the hemantane group (original magnification х100). (c) Periarticular tissue of the ankle joint in the hemantane group (original magnification х100)

 Morphological signs of alteration, destruction and inflammation of the articular cartilage, synovial membrane and periarticular tissue were much less pronounced in the hemantane group than in the AA group. In most cases, the articular surface of the cartilage had sharp boundaries, the same thickness throughout, and a flat surface. The zoning was more clearly defined compared to the AA group. Flat hyperchromic chondrocytes of the superficial zone of the hyaline cartilage were tightly packed in the intercellular substance. Chondrocytes of the deep zone in most cases formed characteristic columns. Chondrocytes of the calcified zone formed small chaotically scattered groups. Cartilage destruction was markedly less pronounced compared to the AA group (Figure 8a). Synovial hyperplasia or proliferation of blood vessels and capillaries in it were not observed. Synovial cells were densely packed, sometimes in several layers (Figure 8b). The periarticular tissue in most cases had the usual microscopic structure without signs of alteration or inflammation and was practically identical to the control group (Figure 8c).

 Morphological signs of alteration, destruction and inflammation were less pronounced in the diclofenac group than in the AA group, but more pronounced than in the hemantane group.

**Figure 9:**
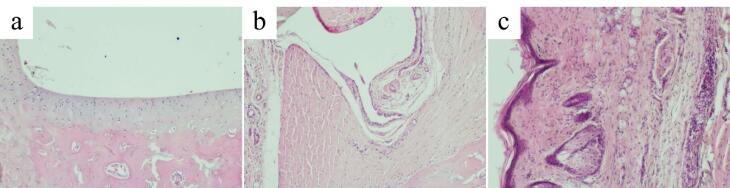
Articular cartilage, synovial membrane and periarticular tissues of rats in the diclofenac group. (a) Articular cartilage of the ankle joint in the diclofenac group (original magnification х100). (b) Synovial membrane of the ankle joint in the diclofenac group (original magnification х100). (c) Periarticular tissue of the ankle joint in the diclofenac group (original magnification х100)

 In most cases, the articular surface of the cartilage had distinct boundaries, the same thickness throughout, and a flat surface. However, the thickness of the articular cartilage was markedly reduced. The characteristic zonal structure was barely visible. Solitarily hyperchromic chondrocytes of the superficial layer of the hyaline cartilage lay in the intercellular substance. Chondrocytes of the deep zone often did not form columns. Chondrocytes of the calcified zone were chaotically dispersed. Metachromasia of the articular cartilage was often observed (Figure 9a). In most cases, rats treated with diclofenac exhibited synovial hyperplasia with proliferation of blood vessels and capillaries (Figure 9b).

 Periarticular tissue in some cases had the usual microscopic structure. However, there were cases of diffuse and focal lymphocytic-macrophagal and single polymorphonuclear leukocyte infiltration of periarticular tissues (Figure 9c).

## Discussion

 This study shows that the noncompetitive low-affinity NMDA receptor antagonist hemantane in a topical formulation reduces the severity of T-cell-mediated autoimmune arthritis induced in rats by an injection of FCA^18^ into a hind paw, which is manifested by edema of the contralateral hind paw. Our data are consistent with the results of Lindblad and colleagues^14^ who showed that blockade of the NMDA receptor channel with another noncompetitive low-affinity NMDA receptor antagonist memantine reduced synovitis and the incidence of erosions in mice with collagen-induced arthritis. Memantine administered intraperitoneally to mice with collagen-induced arthritis induced up-regulation of Foxp3 expression in spleen CD4 + T-cells, followed by an increase in the CD4 + CD25 + Foxp3 + T cell population that suggests peripheral rather than thymic conversion of regulatory T cells.^14^ Adaptive or induced CD4 + CD25 + Foxp3 + regulatory T cells stem from mature CD4 + CD25-Foxp3-precursors at the periphery following adequate stimulation and are physiological actors of the mucosal immune system suppressing immune activation.^19,20^ Subcutaneous injection of another NMDA receptor antagonist, ketamine, prior to intravenous lipopolysaccharide injection suppresses lipopolysaccharide-induced Tumor necrosis factor alpha (TNF-α) production in thioglycolate-elicited macrophages and in the serum of thioglycolate-pretreated mice.^21^ Besides, ketamine inhibits the lipopolysaccharide-induced production of TNF-α, interleukin (IL)-6, and IL-8 in human whole blood^22^ and lipopolysaccharide-induced production of TNF-α in mixed glial cells, astrocyte cultures and microglial cultures.^23^ NMDA receptor antagonist AP-7 enhances the anti-inflammatory effect of dexamethasone in rats with arthritis induced by intra-articular FCA injection.^24^

 In the present study in rats with FCA-induced arthritis, the anti-inflammatory effect of the noncompetitive low-affinity NMDA receptor antagonist hemantane in a topical formulation (5% gel) was non-inferior to the COX inhibitor diclofenac in a topical formulation (1% gel). Both drugs significantly reduced edema of the hind paws in rats with AA after one week of daily therapy. In spite of the hind paw edema in all groups of rats with AA improving on day 28 after the FCA injection, histological examination of the ankle joint of the AA group rats revealed degenerative-dystrophic lesions of the cartilaginous tissue, proliferative inflammation of the synovium, edema and lymphocytic/macrophage infiltration of periarticular tissues. Hemantane gel reduced the severity of degenerative-dystrophic lesions of cartilage tissue better than diclofenac gel.

 Our previous data revealed an anti-exudative effect of 5% hemantane gel in models of acute inflammation, i.e. in rats with paw edema induced by carrageenan or dextran sulfate.^25^ A two-week course of daily treatment with 5% hemantane gel administered topically to the hind paw of rats starting one day before FCA injection into the hind paw reduced paw edema and was non-inferior to intraperitoneal hemantane (10 mg/kg) or diclofenac sodium (5 mg/kg) while showing a more pronounced effect than 1% diclofenac gel.^26^ In acetic acid-induced peritonitis in mice, intraperitoneal injections with hemantane reduced increased phospholipase A2 (PLA2) activity but did not affect the level of COX-2.^27^ The PLA2 family consists of Ca2 + -dependent secretory enzymes that trigger various cell-signaling events in different mammalian tissues,^28,29^ therefore the effect of hemantane on PLA2 activity in peritoneal exudates of mice with acetic acid-induced peritonitis could be explained by a decrease in intracellular concentration of Ca2 + due to the blockade of NMDA receptors.^30,31^ Localizations of NMDA receptors include the central and peripheral nervous system,^32^ in particular in the enteric nervous system,^33,34^ but also lymphocytes. Lymphocytes from rodent species express NMDA-activated iGluR NR1 sub-type, and binding of NMDA to the rodent iGluR NR1 receptor elevates intracellular Ca2 + levels, which can lead to an increase in intracellular reactive oxygen species (ROS) levels and caspase-3 activation.^35^ NMDA receptors expressed by human T-lymphocytes are functionally active in controlling cell activation^36^ and are rapidly up-regulated upon CD4 + T cell activation.^37^

 In the present study, hemantane gel reduced motor deficits and hyperalgesia in rats with FCA-induced autoimmune inflammation. In the focus of inflammation, high-threshold nociceptors (A-delta and C-fibers) are activated under slight mechanical pressure with the release of glutamate and aspartate. Glutamate and aspartate binding NMDA receptors are localized on unmyelinated axons at the dermal-epidermal junction in the glabrous and hairy skin of rats^9,38^ and in human hairy skin.^10^ Besides, glutamate receptors are transported from the dorsal root ganglion cell bodies into central and peripheral primary afferent terminals.^39^ In sensory neurons, NMDA receptors functionally interact with TRPV1 via CaMKII and PKC signaling cascades,^40^ and an injection of NMDA receptor antagonists into the hind paw of rats reduced the thermal hyperalgesia induced by capsaicin.^41^ Hemantane administered to mice by cutaneous application, intraperitoneally or by subcutaneous injection in the metatarsus reduced the duration of the pain response to subcutaneous injection of TRPV1 receptor agonist capsaicin in the metatarsus.^42^

 On day 14 after the FCA injection (before treatment), plateletcrit, platelet and WBC counts were elevated in all groups of rats with AA. In patients with RA, platelet counts gradually increased with radiological disease progression,^43,44^ which is presumably caused by an up-regulation in megakaryocytopoiesis induced by several pro-inflammatory pleiotropic cytokines, namely IL-11, stem cell factor, leukemia inhibitory factor, IL-6, granulocyte colony-stimulating factor, and thrombopoietin.^45^ WBC count elevation in rats with AA was caused by an increase in segmented neutrophils, which is consistent with clinical data.^46^ Dysregulated neutrophil activation can contribute to RA development and progression by producing ROS, granule proteases, cytokines and chemokines.^47^ On day 28 after the FCA injection, segmented neutrophil percentage (in WBC differential) significantly decreased compared to day 14 in the AA and hemantane group but not in the diclofenac group; plateletcrit, platelet and WBC counts decreased in all rats with AA to the level of animals without inflammation, which is consistent with reduced paw edema of rats with AA.

 On day 28 after the FCA injection, reduced body weight and spleen hypertrophy were registered in rats of the AA group. These data are consistent with the results of studies in rats with FCA-induced arthritis^48,49^ and in particular with our previous data.^26^ Neither hemantane gel nor diclofenac gel had a significant effect on reduced body weight or spleen hypertrophy in rats with AA. Fatalities of rats with AA were only registered in the diclofenac gel group (4 rats out of 10). This result reproduces our previous findings,^50^ the only difference being that diclofenac gel was previously applied daily starting one day before the FCA injection. Notably, in the previous study, no fatalities among rats with AA were observed in the group that received daily intraperitoneal injections of 5 mg/kg diclofenac sodium for 14 days. Diclofenac sodium administered orally at the same daily dose for 21 days caused the death of 4 out of 10 rats due to enteropathy, which was identified by iron-deficiency anemia, thinning of the gastric and intestinal walls, bile reflux in the stomach, and almost no food residue in the gastrointestinal tract.^50^ In the present study, rats treated with gels applied locally to the hind paws exhibited no such signs.

 NMDA receptor antagonists administered orally or parenterally affect the central nervous system, inducing such side effects as psychosis, memory impairment, anesthesia, and neuronal cell death, which limits their clinical usefulness for chronic conditions.^51-53^ Although amantadine (5 mg/kg orally daily for three weeks) improved owner-reported impaired mobility and owner-perceived quality of life in cats with osteoarthritis, the drug significantly decreased the activity of animals.^54^ No such side effects were observed in our studies of hemantane gel. In the present study, hemantane gel completely eliminated FCA-induced motor deficits in rats. In the previous study, hemantane gel improved horizontal movement in rats, which had decreased due to FCA injection, as indicated by actometry.^55^

## Conclusion

 To summarize, we have demonstrated that the noncompetitive low-affinity NMDA receptor antagonist N-(2-adamantyl)-hexamethyleneimine hydrochloride (hemantane) in a topical formulation (5% hemantane gel) applied locally to hind paws daily alleviates FCA-induced arthritis in rats, namely edema of the hind paws, hind paw hyperalgesia, motor deficits and histological signs of inflammation of the ankle joint tissues. Its effect is comparable to the COX inhibitor diclofenac in a topical formulation (1% diclofenac gel). These data represent another confirmation of the anti-inflammatory effect of NMDA receptor antagonists, hemantane in particular, and justify further investigation of topical NMDA receptor antagonists as add-on treatments for musculoskeletal conditions.

## Acknowledgments

 We are grateful to the Laboratory of Finished Dosage Forms, FSBI Zakusov Institute of Pharmacology, Moscow for providing 5% hemantane gel.

 This work was supported by State Program for Fundamental Scientific Research N 0521-2019-0007.

## Competing Interests

 Authors have no conflict of interest to declare.

## Ethical Approval

 All animal experimental procedures were conducted in compliance with GOST (Russian National Standard) 33216-2014, “Guidelines for accommodation and care of animals. Species-specific provisions for laboratory rodents and rabbits”; GOST (Russian National Standard) 33215-2014, “Guidelines for accommodation and care of animals. Environment, housing and management”; and Directive 2010/63/EU of the European Parliament and of the Council of 22 September 2010 on the protection of animals used for scientific purposes. The experimental protocol was approved by the Biomedical Ethics Committee, FSBI Zakusov Institute of Pharmacology (Protocol No. 01 of Jan. 31, 2020).
